# Glucagon-like peptide-1 receptor (GLP-1R) overexpression defines a distinct immunogenetic subset in primary and metastatic thyroid cancer: implications for GLP-1R agonist therapy

**DOI:** 10.3389/fonc.2026.1834606

**Published:** 2026-05-28

**Authors:** Sophia Kennedy, Jerin Thomas, Stella D’Arcy, Ivie Odia, Dev Kamdar, Lucio Pereira, Danielle Scarola, Brett Miles, Douglas Frank, Charit Taneja, Nagashree Seetharamu, Rajarsi Mandal

**Affiliations:** 1 Northwell, New Hyde Park, NY, United States; 2 Feinstein Institutes for Medical Research, Northwell Health, Manhasset, NY, United States; 3 Donald and Barbara Zucker School of Medicine at Hofstra/Northwell, Hempstead, NY, United States; 4 Department of Otolaryngology, Long Island Jewish Medical Center, New Hyde Park, NY, United States; 5 Department of Endocrinology, Long Island Jewish Medical Center, New Hyde Park, NY, United States; 6 Department of Medicine, Long Island Jewish Medical Center, New Hyde Park, NY, United States

**Keywords:** GLP-1 receptor agonists, medullary thyroid carcinoma, mRNA expression, papillary thyroid carcinoma, TCGA database

## Abstract

**Introduction:**

The surge in prescribing glucagon-like peptide-1 receptor agonists (GLP-1 RAs) for diabetes and weight management highlights a pressing need to characterize their associated risks. All GLP-1 RAs include an FDA boxed warning for increased risk of development of medullary thyroid carcinoma (MTC) based on early murine studies. However, clinical evidence remains conflicting and highly debated, highlighting the importance of comprehensively characterizing GLP-1R expression in both primary and metastatic thyroid cancers.

**Methods:**

We leveraged transcriptomic data from The Cancer Genome Atlas (TCGA) from 11,160 patients across 33 cancer types to comparatively analyze GLP-1R transcriptomic expression across normal, primary, and metastatic tissues. We also performed flow cytometric analysis on primary and metastatic medullary and papillary thyroid carcinomas (PCT) to quantify differential GLP-1R protein expression levels.

**Results:**

Strikingly, metastatic PTC displayed the highest median GLP-1R mRNA expression among all TCGA cancers. This finding was supported by flow cytometry of patient tumor samples, which confirmed elevated GLP-1R protein expression in both PTC and MTC metastatic tissues as compared to their respective primary tumors. Further, GLP-1R expression levels stratified TCGA thyroid cancers into unique immunogenetic transcriptional profiles. Tumors with high GLP-1R expression were notably associated with downregulated immune pathway activity and decreased immune cell infiltration.

**Discussion:**

These findings identify a subset of thyroid carcinomas with high GLP-1R expression, most pronounced in metastatic disease, which are accompanied by distinct genetic and immunological profiles. As GLP-1R agonist therapies continue to expand in clinical use, further investigation is warranted to determine the oncogenic implications of this overexpression for thyroid cancer.

## Introduction

Glucagon-like peptide-1 (GLP-1) receptor agonists (RAs) (e.g., Semaglutide (Ozempic) and Tirzepatide (Mounjaro)) have historically been prescribed to treat Type-2 Diabetes Mellitus (T2DM), but their efficacy in managing weight loss has increased their role in weight management treatments (1, 2). GLP-1 RAs simulate the function of GLP-1 in the body to promote the release of insulin, lowering blood-glucose, thereby promoting satiety (1). Beyond their intended effect for T2DM, GLP-1 RAs can also improve overall metabolic and neurological health, decreasing risk for cardiovascular events, reducing cholesterol, and diminishing the risk of developing Alzheimer’s and Parkinson’s Disease by treating insulin resistance (1, 3, 4). Given their ability to manage obesity and promote weight loss, GLP-1 RA usage has increased rapidly over the last decade. A recent poll found that 18% of adults reported taking a GLP-1 RA in their lifetime, an increase from the previous year’s poll, which cited 12% of Americans reported GLP-1 RA use in their lifetime (5, 6). The majority of these adults indicated they began taking GLP-1 RAs to manage metabolic or cardiovascular disorders, though a remaining 40% of users reported they were using the drugs for weight loss (5).

At present, the risk of prolonged GLP-1 RA usage as it pertains to the thyroid and the development of medullary thyroid carcinoma (MTC) is unclear. MTC is a rare cancer of the parafollicular cells, or C-cells, in the thyroid, with around 1,000 new cases diagnosed in the United States each year (7, 8). Parafollicular cells, which comprise 2-4% of thyroid tissue, are involved in the physiologic regulation of calcium levels through production of calcitonin (8–10). Current prescriptions for GLP-1 RAs include boxed warnings for increased risk of development of MTC (11). This warning is based on a dose-dependent rodent trial that found that mice exposed to supraphysiologic doses of GLP-1 RAs were more likely to experience C-cell hyperplasia (12, 13). However, they also concluded that this increase in C-cells mediated by GLP-1 RA exposure was not correlated with RET activation, a developmental pathway of medullary thyroid cancer (12).

The pathway mechanisms that underpin GLP-1R agonism-induced C-cell hyperplasia are not well understood, however some have connected GLP-1 RA-related C-cell effects with mTOR activation (12). GLP-1R is a G-protein coupled receptor that activates that phospho-PI3K-Akt pathway in the pancreas, promoting pancreatic β-cell function (14). Akt can activate mTORC1, which could lead to cell proliferation and is implicated in the development of many human cancers (14, 15). While there is a diminished risk of MTC development so long as GLP-1 RA use is limited to dosages approved by the FDA, prolonged usage of GLP-1 RAs (1 to 3 years) has been associated with an increased risk for the development of all thyroid carcinomas (16).

MTC has a poor prognosis, and while representing less than 5% of thyroid carcinomas, it is responsible for 13% of thyroid carcinoma deaths (17). MTC is not always easily detected early in disease progression, and the five-year survival for advanced stage MTC is 28% (7). MTC is primarily a sporadic cancer, though it can be hereditary; common risk factors for inherited MTC are familial history of MTC and hereditary MEN2 syndrome (7, 18). The wide array of conflicting data on GLP-1R agonism and related downstream signaling as it pertains to MTC development necessitates the continued investigation into these mechanisms. GLP-1R expression across primary and metastatic thyroid cancers has not been comprehensively characterized. Here, we aim to profile GLP-1R protein and mRNA expression in primary and metastatic thyroid cancers to explore a potential link between GLP-1R agonism and thyroid oncogenesis.

## Materials and methods

### Data acquisition and analysis

Transcriptome Profiles for all 33 human cancers in The Cancer Genome Atlas (TCGA) were acquired from the Genomic Data Commons (GDC) and analyzed in R (V.4.4.1). TCGA is a publicly available deidentified online cancer database, therefore no prior authorization is required. Pan-cancer data was normalized utilizing the R package DESeq2’s Median of Ratios Normalization (19). We visualized a bimodal distribution among TCGA thyroid sample GLP-1R expression data, and divided samples accordingly along this division such that samples in the high group were approximately in the top 25% of mRNA expression level (expression ≥ 300) and samples in the low group were approximately in the bottom 75% (expression < 300). Data visualization was performed using clusterProfiler, EnhancedVolcano, ggplot, and DOSE packages (20–23). Gene Set Enrichment Analysis was performed using the clusterProfiler and fgsea packages (20, 24). Two-dimensional reduction visualization based on over 36,000 gene transcripts were created using the online interactive TumorMaps portal and stratified by GLP-1R expression level (25).

Immune deconvolution data was downloaded from the TIMER2.0 database under the filename “Infiltration Estimation,” (26). quanTIseq (27), CIBERSORT (28), and MCP-counter (29) data for primary and metastatic TCGA-THCA samples (n = 509) was log10 transformed and subsequently analyzed. Heatmaps of gene expression were created using the pheatmap R package (30).

### Institutional review board approval

This study was approved by the Northwell Health Institutional Review Board (IRB #24-0335). Human thyroid carcinoma tumor specimens (**Table 1**) were collected from primary and metastatic tissue of consenting patients in accordance with our IRB-consented protocol (IRB #24-0335). Patients provided written informed consent prior to collection. Specimens were de-identified and subsequently processed.

**Table 1:** Clinical background for thyroid carcinoma patients.

Patient #	Cancer type	pT	pN
20	Medullary Thyroid Carcinoma	pT3a	pN1b
24	Medullary Thyroid Carcinoma	pT1b	pN1b
32	Papillary Thyroid Carcinoma	pT3a	pN1b

### Human tumor samples processing

Specimens were minced into 0.5mm^3^ pieces and dissociated into single cell suspensions using the Human Tumor Dissociation Kit (Miltenyi) and GentleMACS Octo-Dissociator with Heaters (Miltenyi). Dissociated cells were strained using a 70uM strainer, spun down for five minutes, resuspended and counted, washed with PBS and spun again. Pellets were then resuspended in freezing media consisting of 40% DMEM, 10% DMSO, and 50% FBS, and aliquoted into barcoded cryovials, and cryopreserved.

### PANC-1 cell line

The PANC-1 (pancreatic ductal carcinoma) cell line was purchased from the American Type Culture Collection (ATCC), carefully thawed, and cultured in DMEM (Gibco) with 1:100 Penicillin/Streptomycin (Gibco) and 10% FBS (Gibco).

### Flow cytometry

Cryopreserved primary human tumor specimens (*n* = 3) were used for flow cytometric analysis. Cells were thawed slowly and resuspended in warmed DMEM (Gibco) supplemented with 10% FBS (Gibco) and 1X Penicillin Streptomycin (Gibco). Cells were then rinsed with phosphate-buffered saline (PBS) and blocked with Human TruStain FcX (Biolegend). A live/dead stain was performed with Zombie Aqua Fixable Viability Dye (Biolegend) for 20 minutes. Cells were fixed and permeabilized using the CytoFast Fix/Perm kit (Biolegend) per manufacturer’s instructions. Cells were stained with fluorophore conjugated antibodies (CD3-BUV737 (BD Biosciences; Clone: SK7; 5uL), CHGA-BV421 (BD Biosciences; Clone: S21-537; 5uL), and GLP-1R-PE (R&D Systems; Clone: 197920; 5uL)) for 20 minutes at RT in the dark. Following staining, cells were acquired on a BD FACS Symphony and analyzed using FlowJo V10. Threshold for GLP-1R-positvity was established using flow cytometry read outs from the PANC-1 cell line (**Supplementary Figure 1**).

## Results

**Figure 1:**
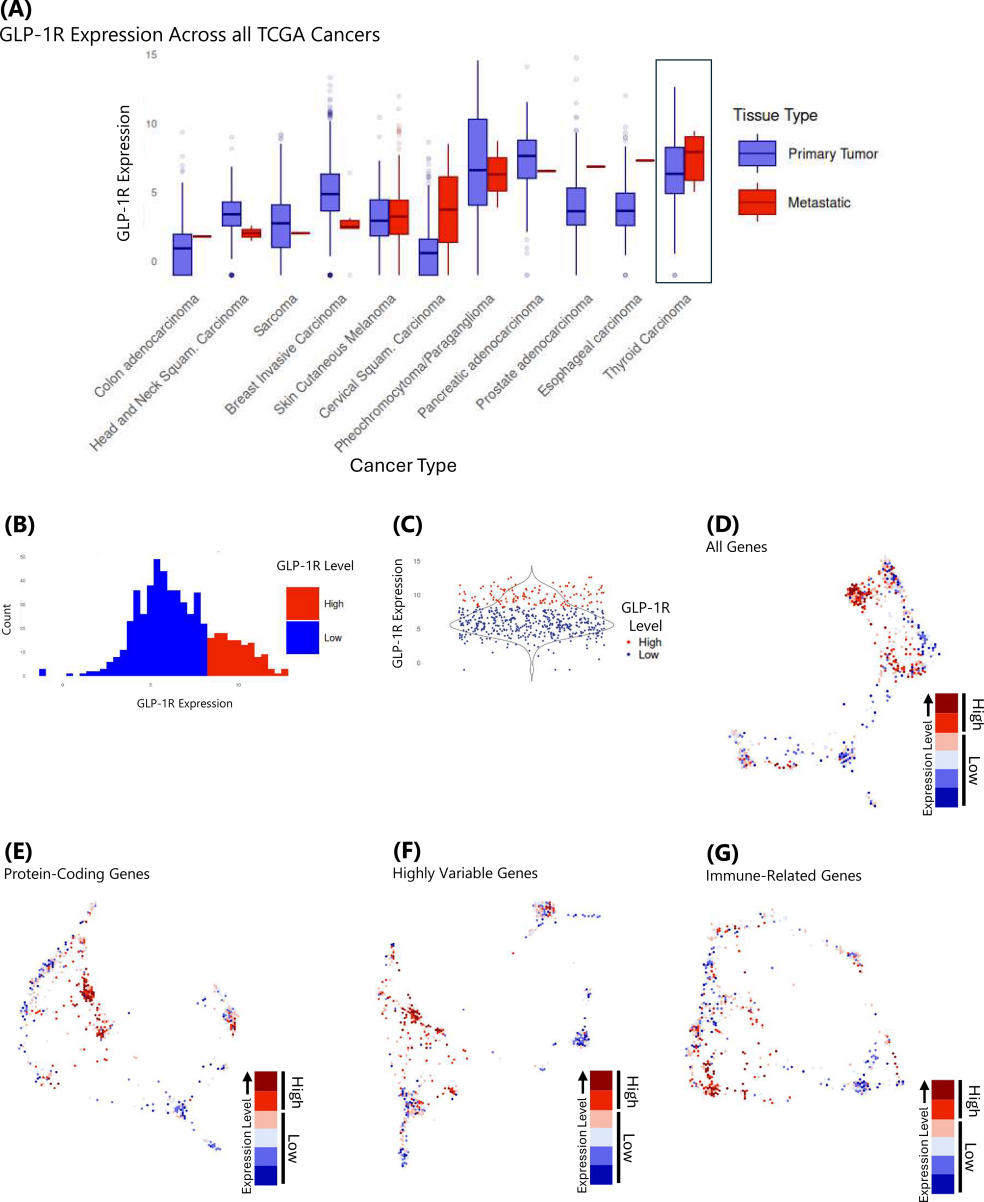
Metastatic thyroid carcinoma tissue shows highest GLP-1R expression across all cancer types in TCGA. Distinct populations of high versus low expression are evident in thyroid carcinoma patient samples. **(A)** Boxplots expressing GLP-1R expression quartiles for primary and metastatic tissue from TCGA database. **(B)** Histogram indicating division between high and low GLP1R expression samples in TCGA Thyroid Carcinoma subset. **(C)** Violin plot demonstrating separation between high and low populations in thyroid carcinomas. **(D)** TumorMap displaying arrangement of TCGA Thyroid Carcinoma patients stratified by GLP-1R expression level for all genes (36,327 genes), **(E)** protein coding genes (18,713 genes), **(F)** highly variable genes (2,000 genes), and **(G)** immune related genes (2,565 genes).

Using data obtained from The Cancer Genome Atlas (TCGA), median GLP-1R expression was compared across all TCGA cancer types, split by primary and metastatic tissue, and found to be highest in metastatic papillary thyroid carcinoma (**Figure 1a**), despite normal thyroid tissue being ranked 6^th^ highest overall among TCGA cancers (**Supplementary Figure 2**). Using TCGA Thyroid Carcinoma samples, (*n* = 572), we identified a clear division of populations between samples with high GLP-1R expression and samples with low GLP-1R (**Figures 1b, c**). Within all thyroid samples, high expression was determined to be any mRNA expression greater than 300 (approximately top 25^th^ percentile of samples). Low expression was any mRNA expression less than that (approximately bottom 75^th^ percentile of samples). Using TumorMap’s two-dimensional reduction visualization, we aimed to understand whether GLP-1R expression led to transcriptomic grouping of high versus low samples (25). Importantly, we found that GLP-1R high samples clustered together relatively strongly across all coding and non-coding genes (36,327 genes), suggesting overarching trends in tumor biology and underlying transcriptomic similarities (**Figure 1d**). Further, this trend was similarly observed in three distinct genetic subsets: in only protein-coding genes (18,713 genes) (**Figure 1e**), in the highest variable genes (2,000 genes) (**Figure 1f**), and in a set of previously identified (31) pan-immune-related genes (2,565 genes) (**Figure 1g**). This division speaks to the possibility of distinct clusters of tumor environments, in part differentiated by GLP-1R expression level.

**Figure 2:**
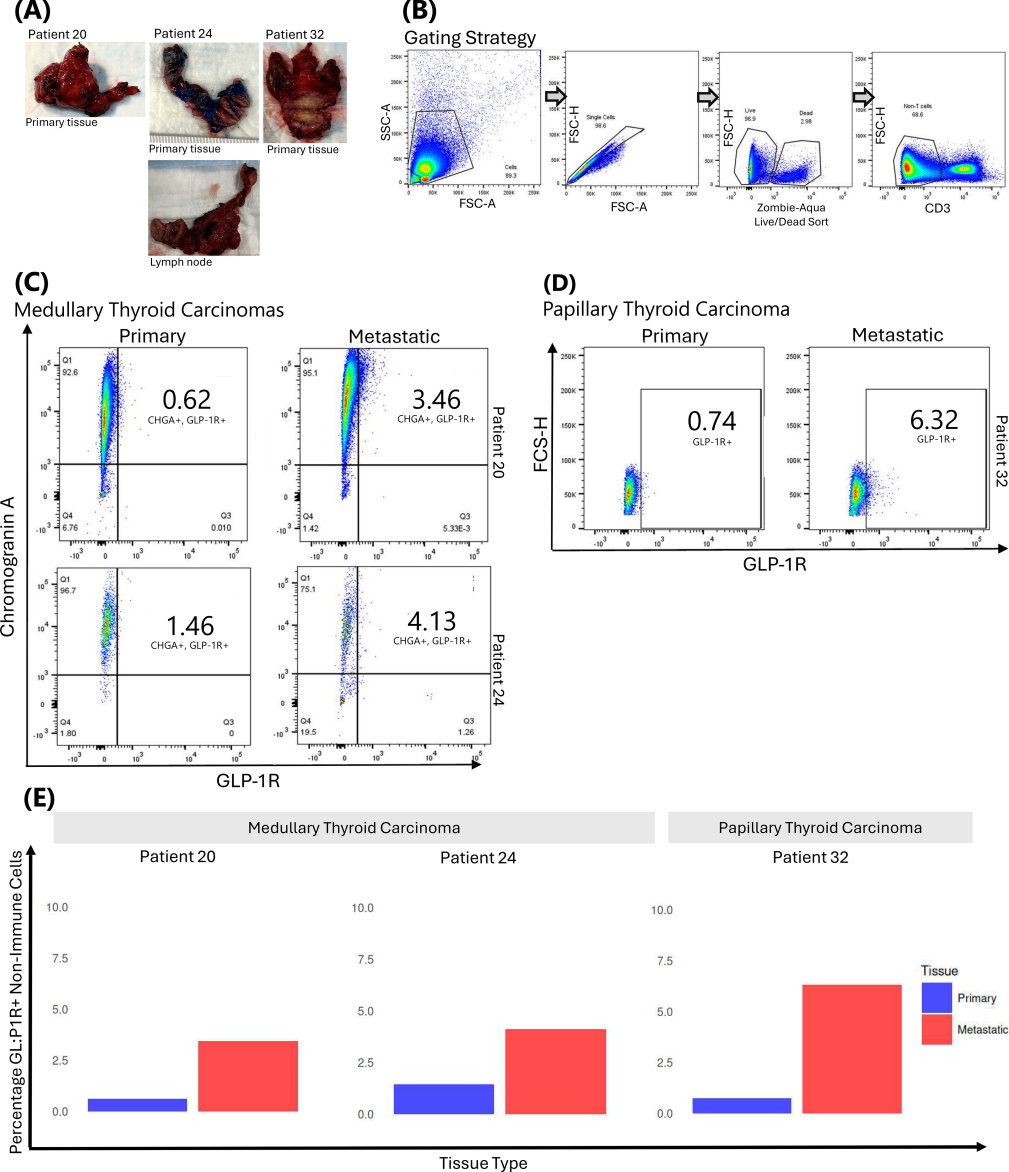
Flow cytometry analysis of GLP-1R expression in papillary and medullary thyroid carcinoma patients. **(A)** Photos of tumor tissue specimens following surgical removal from consenting patients. **(B)** Flow cytometry gating strategy for each patient. Following removal of debris, singlets are isolated and subsequently segregated on viability and CD3 status. **(C)** Quadrant-style gates of CHGA and GLP-1R antibody-binding for isolated non-T cells of primary and metastatic medullary thyroid carcinoma patients 20 and 24. **(D)** GLP-1R antibody-binding for isolated non-T cells of primary and metastatic papillary thyroid carcinoma patient 32. **(E)** Bar plots indicating change in percentage of GLP-1R+ cells between primary and metastatic tissue for all three patients.

Primary patient tumor samples (**Figure 2a**) were analyzed via flow cytometry to examine differences in GLP-1R protein levels between primary and metastatic tumors. The general gating strategy is shown in **Figure 2b**. Positive and negative GLP-1R cutoffs were established using a PANC-1 cell line (**Supplementary Figure 1**). This cell line is known to express GLP-1R and was therefore used as a positive GLP-1R control. GLP-1R positive and negative gates were made using PANC-1 flow read outs as a guide. We found a marked increase in the percentage of chromogranin A (CHGA+), a parafollicular C-cell marker, and GLP-1R double-positive cells as disease progressed from primary to metastatic (**Figure 2c**). Further analysis revealed that the CD3- compartment (non-T cell compartment) in PTC patient 32 followed a similar pattern, with the percentage of GLP-1R+ cells increasing in metastatic tissue compared to primary tissue (**Figure 2d**). Across all three patients, there is a notable and consistent increase in the proportion of GLP-1R+ cells in the metastatic sample as compared to the primary (**Figure 2e**).

**Figure 3:**
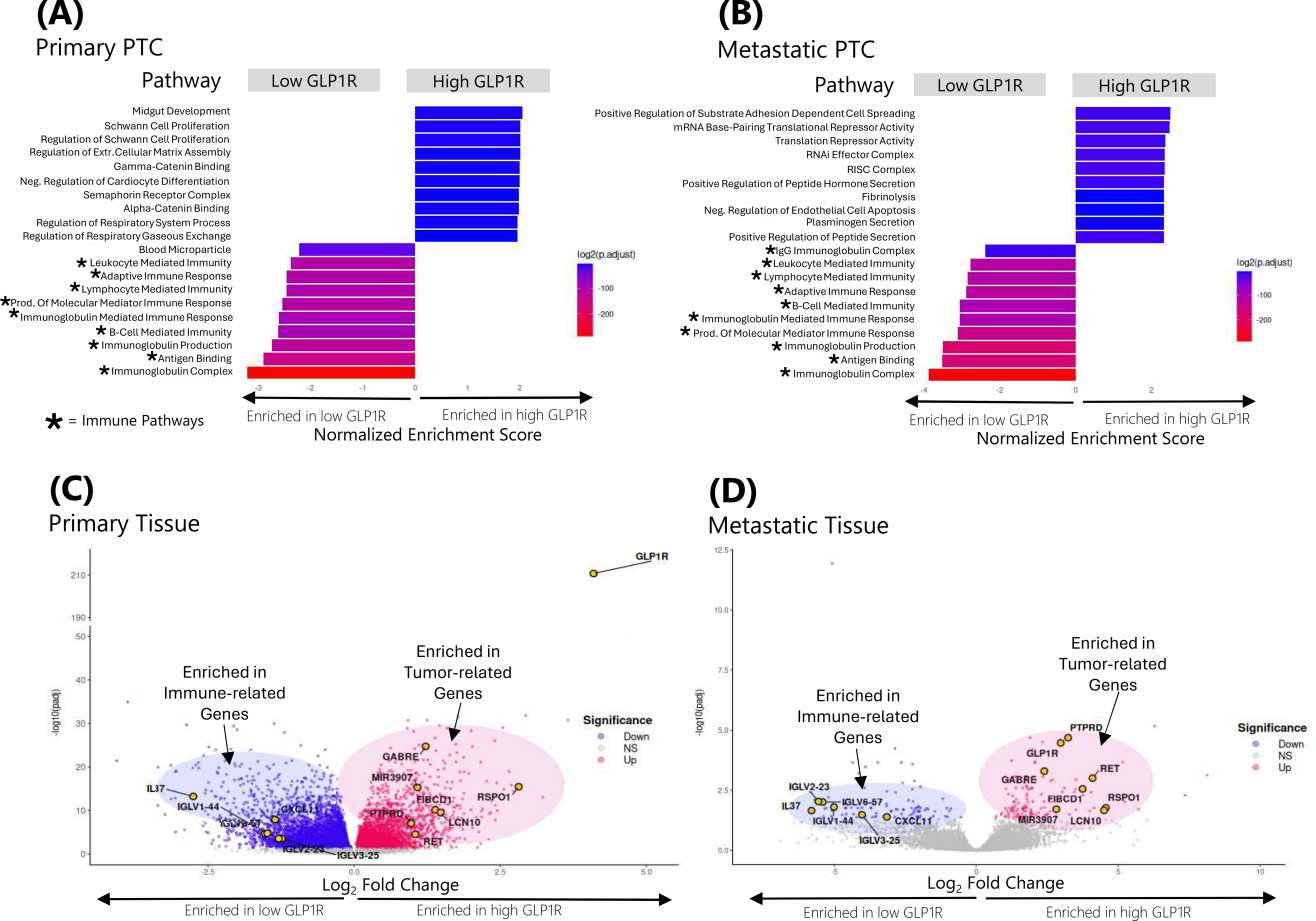
GSEA of high and low GLP-1R expression populations in TCGA thyroid carcinoma primary and metastatic tissue. **(A)** Bar plot shows the highest enriched pathways in primary PTC, split by GLP-1R expression level (low versus high). A positive Enrichment Score indicates the pathway is enriched in the *GLP-1R-High* group relative to the *GLP-1R-Low* group, and vice versa for the negative enrichment score. Bar color correlates to a log_2_ adjusted p-value. **(B)** Bar plot shows the highest enriched pathways in metastatic PTC, split by GLP-1R expression level (low versus high). A positive Enrichment Score indicates the pathway is enriched in the *GLP-1R-High* group relative to the *GLP-1R-Low* group, and vice versa for the negative enrichment score. Bar color correlates to a log_2_ adjusted p-value. **(C)** Volcano plot shows the log_10_ adjusted p-value over the fold change of all genes for high- and low-GLP-1R expressing PTC patient samples in primary tissue and **(D)** metastatic tissue. Genes identified in **Supplementary Tables 1** and **2** are highlighted. Genes with negative log_2_ fold change are enriched in *GLP-1R-Low* samples; genes with positive log_2_ fold change are enriched in *GLP-1R-High* samples.

Differential Gene Expression (DGE) was performed, and subsequent Gene Set Enrichment Analysis (GSEA) was executed on DGE results to better understand pathway enrichment discrepancies between sample types. Samples were grouped by tissue type (primary or metastatic) and again by GLP-1R expression level (high or low). GSEA revealed clear differences in immune pathway enrichment between populations. Within all primary thyroid carcinoma samples, *GLP-1R-low* samples had increased immune-related pathway enrichment when compared to *GLP-1R-high* samples (**Figure 3a**). Similarly, metastatic thyroid tissue samples in the *GLP-1R-low* category had increased immune-related pathway enrichment when compared to samples in the *GLP-1R-high* category (**Figure 3b**).

In an effort to better understand gene profiles between high and low GLP-1R groups, we examined overlapping upregulated genes between primary and metastatic tissue in both expression groups. Samples with *GLP-1R-high* levels in both primary and metastatic tissue were enriched in genes involved in proliferation, tumor development, and multiple prognostic genes associated with other cancers (**Supplementary Figure 3a**; **Supplementary Table 1**). Conversely, we observed that primary and metastatic tissue in the *GLP-1R-low* group shared several immune-related genes (**Supplementary Figure 3b**; **Supplementary Table 2**). Volcano plots (**Figures 3c, d**) were constructed to highlight differentially expressed genes (DEGs) between both comparisons, and genes that overlapped between primary and metastatic tissue were highlighted.

**Figure 4:**
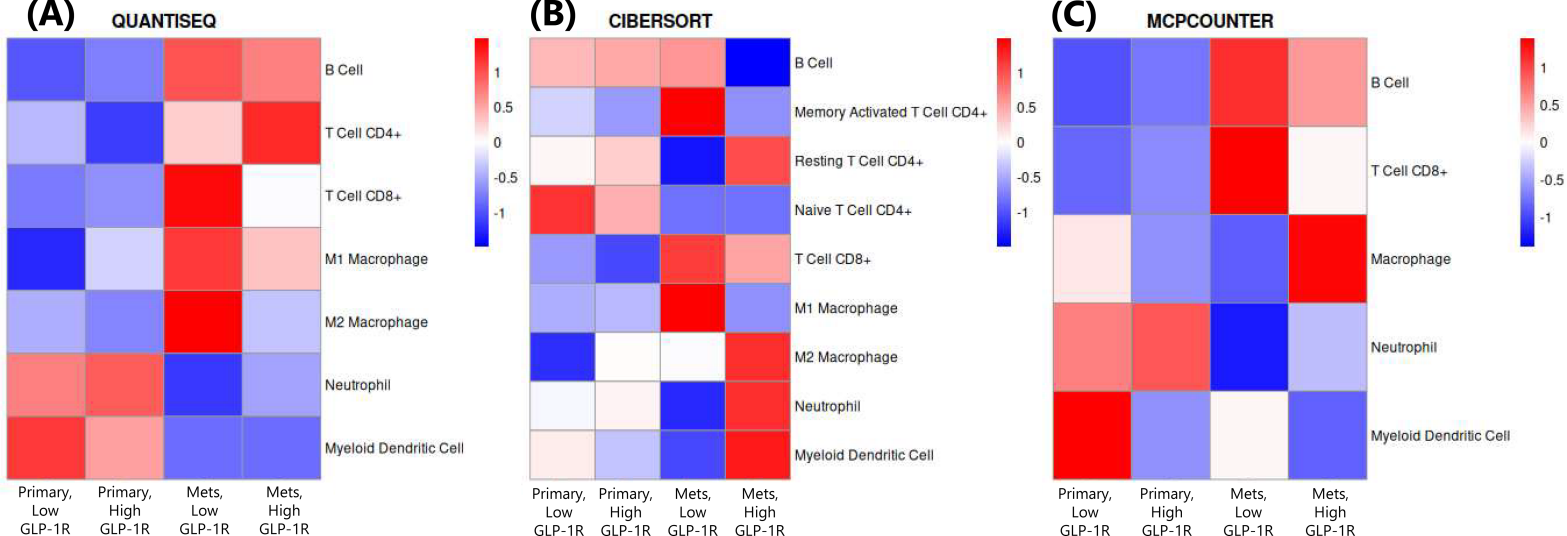
Immune cell enrichment from multiple immune deconvolution analyses. Heatmaps of log_10_ transformed and scaled immune enrichment data for selected immune cell types from three immune deconvolution methods: **(A)** quanTIseq, **(B)** CIBERSORT, and **(C)** MCPCounter.

To better understand how immune cell type populations change as disease progresses from primary to metastatic for *GLP-1R-high* and *GLP-1R-low* patients, we leveraged data from the TIMER2.0 database and examined changes in immune cell type enrichment across tissue and GLP-1R level for three different Immune Deconvolution Methods: quanTIseq (27), CIBERSORT (28), and MCP-counter (29) (**Figures 4a–c**). For primary tissue, all three deconvolution methods reflected lower Myeloid Dendritic Cell enrichment in *GLP-1R-high* patients compared to *GLP-1R-low* (**Figures 4a–c**). Additionally, CIBERSORT data showed lower CD8+ T Cell levels in primary *GLP-1R-High* compared to *GLP-1R-Low* (**Figure 4b**). In metastatic tissue, we observed a decrease in B Cell enrichment in *GLP-1R-high* tissue compared to *GLP-1R-low* tissue in all three methods, as well (**Figures 4a-c**). In the CIBERSORT dataset, memory activated CD4+ T cells decreased in *GLP-1R-high* primary and metastatic tissue when compared to both *GLP-1R-low* groups, while resting CD4+ T cells increased in the *GLP-1R-high* groups (**Figure 4b**). For all three deconvolution methods, CD8+ T cells were also observed to decrease in the *GLP-1R-high* metastatic tissue when compared to that of *GLP-1R-low* tissue (**Figures 4a–c**).

## Discussion

With millions of Americans prescribed and taking GLP-1 RAs, a clear understanding of the long-term risk of GLP-1R agonism as it pertains to the thyroid is critical. While some studies do not identify an increase in the development of thyroid carcinomas (32), continued investigation into the effects of sustained GLP-1RA exposure is paramount for broadening our depth of understanding the potential risks associated with these therapies. The lack of clarity surrounding the risk of associated thyroid carcinoma development demonstrates a clear gap in the literature and a necessity to better derive these mechanisms.

The computational findings of this study demonstrate a prominent increase in GLP-1R expression in metastatic thyroid carcinoma tissue when compared to all other TCGA cancer tissues. Following stratification of high and low GLP-1R expression, we found distinct differences in the transcriptional and immunologic landscapes, highlighting an underlying biologically driven pattern between these two groups. Flow cytometry read outs from patient tumor samples support our computational findings, showing an increase in GLP-1R+ cell percentages in metastatic tissues in both papillary and medullary carcinomas. The increase in GLP-1R positivity in metastatic cells raises potential questions about the role of GLP-1R in thyroid carcinoma disease progression. Further analysis illuminates two distinct tumor environments in both metastatic and primary thyroid carcinoma patients on the basis of GLP-1R expression. This finding offers novel insight into thyroid carcinoma patient demographics and new potential for possible prognosticator exploration. Additionally, these observations raise questions about the differences in GLP-1R activation in metastatic disease compared to primary disease.

Based on these findings, we explored differences between GLP-1R expression groups at both a pathway and a cellular level. GSEA findings revealed immune pathway activity unregulated in the *GLP-1R-low* group and suppressed in the *GLP-1R-high* group. Further exploration using DGE analysis indicated that *GLP-1R-high* groups in both tissue types were similarly enriched in genes that code for cell proliferation (33, 34), genes implicated in tumor development (35), and prognosticator genes (36–39). Conversely, *GLP-1R-low* groups in both tissue types were enriched in genes that code for interleukin-37, a cytokine protective against some cancers (40), and multiple immunoglobulins. These differences in cell type enrichment pose important questions about how GLP-1R expression level affects cancer pathology and whether GLP-1 RAs may exacerbate these phenomena.

This study’s discovery of *GLP-1R-high* samples displaying suppressed immune-related pathways prompted further exploration utilizing three different immune cell deconvolution methods. Heatmap results revealed decreasing immune cell enrichment in *GLP-1R-high* samples when compared to *GLP-1R-low* samples across these methods for a selection of key immune cells, further validating the phenomenon of *GLP-1R-high* samples having immune-cold tumor microenvironments. This difference in immune activity between primary and metastatic tissues on the basis of GLP-1R expression could be indicative of GLP-1R-induced immunosuppressive activity in the tumor environment. Given the severity of late-stage thyroid carcinoma, this finding suggests a possible pathway for GLP-1 RA-associated thyroid carcinoma progression and mechanism. One study was able to identify a relationship between a GLP-1RA and M1 macrophage polarization, alleging an antitumor effect from Semaglutide exposure (41). While this study was limited to murine models, the results point to the presence of GLP-1RA-induced immune modulation. Further studies should investigate the role of GLP-1RAs in metastatic tumor immune microenvironments.

GSEA immune pathway discrepancies between high and low GLP-1R-expressing samples, and subsequent immune cell deconvolution analysis, demonstrated a decrease in immune cell density in *GLP-1R-high* samples. Decreased immune activity in the tumor microenvironment could contribute to poor prognosis and an inability to mitigate tumor growth, potentially due to immunosuppression by cancer cells or other immunosuppressive populations in the tumor microenvironment (42). While a similar trend was observed between tissue type, with metastatic tissue having decreased immune-cell infiltration when compared to primary tissue, immune cell type enrichment consistently differed between primary and metastatic comparisons and high and low GLP-1R comparisons, suggesting a subgroup-specific pattern of immune modulation. Further analysis of the tumor microenvironment could help explain how GLP-1R expression functions with respect to tumor immune infiltration.

Our study is subject to several limitations. The TCGA database only includes transcriptomic data for thyroid carcinomas from PTC patients, not MTC. Transcriptomic data for MTC is scarce due to its relative rarity, and future studies may help close this gap. It is important to note that conclusions drawn from transcriptomic PTC data from TCGA-THCA cannot be directly translated to MTC and transcriptomic MTC data are needed to investigate if the findings in this study can be similarly observed in a large-scale MTC dataset. Additionally, the TCGA database does not disclose whether patients are GLP-1 RA-exposed or -naïve, thus future transcriptomic studies may comparatively investigate these patient populations across cancer types to further dissect immunogenomic differences. Further, the small cohort of PTC and MTC patient tumor samples available for our flow cytometry analyses introduced an additional limitation to the study; patient tumor samples are scarce, and we were limited by our number of precious samples. Future studies may seek to include a higher volume of patient tumor samples, should they be available. While we were able to highlight interesting patterns and phenomena in both the TCGA-THCA computational cohort and in our MTC and PTC patient samples analyzed with flow cytometry, more studies are needed in order to understand relationship between the increase in GLP-1R expression in thyroid carcinomas and any changes in immune infiltration before causality can be established. Mechanistic studies need to be performed to understand what drives GLP-1R upregulation and the increase in pro-tumorigenic transcription.

This study demonstrates elevated GLP-1R levels in metastatic thyroid cancers compared to other TCGA cancers, with distinct *GLP-1R-high* and *GLP-1R-low* expression profiles correlating with immune pathway activation. These findings suggest a possible role for exogenous GLP-1R agonism on primary and metastatic thyroid tissues warranting further investigation. Interestingly, a retrospective observational cohort study on 18 patients with PTC did identify two patients whose tumor volume increased following exposure to GLP-1RAs (43); though, the majority of patients saw no significant volume change. However, the lack of patients with MTC is a limitation of this study. With the widespread and rapidly expanding use of GLP-1 RAs, it is paramount to further solidify our understanding of the potential proliferative effects of GLP-1R signaling in the thyroid as it pertains to the development of thyroid cancer.

## Data Availability

Publicly available datasets were analyzed in this study. This data can be found here: https://portal.gdc.cancer.gov/.

## Supplementary material

Supplementary Figure 1 Threshold for GLP-1R-positivity in flow cytometry. Following removal of debris, singlets are isolated and subsequently gated on viability. GLP-1R-positivity was established on the basis of fluorescence relative to a non-anti-GLP-1R labelled sample.

Supplementary Figure 2 GLP-1R expression across all TCGA normal tissue. Expression is normalized and log_2_-scaled.

Supplementary Figure 3 Venn diagrams display all significantly enriched (adjusted p-value ≤ 0.05) genes for primary and metastatic tissue for **(A)**. *GLP-1R-High* and **(B)**. *GLP-1R-Low* samples from DESeq differential gene expression analysis.

Supplementary Table 1 Overlapping genes upregulated in high GLP-1R primary and metastatic tissue.

Supplementary Table 2 Overlapping genes upregulated in low GLP-1R primary and metastatic tissue.
