# Supplementary material for: Glucagon-like peptide-1 receptor (GLP-1R) overexpression defines a distinct immunogenetic subset in primary and metastatic thyroid cancer: implications for GLP-1R agonist therapy
Source: Front Oncol. 2026 May 28;16:1834606. doi: 10.3389/fonc.2026.1834606 (PMC13253391; doi:10.3389/fonc.2026.1834606)
Supplement: Supplementary Figure 1 — Threshold for GLP-1R-positivity in flow cytometry. Following removal of debris, singlets are isolated and subsequently gated on viability. GLP-1R-positivity was established on the basis of fluorescence relative to a non-anti-GLP-1R labelled sample. [file Presentation1.pptx]

## Slide 1
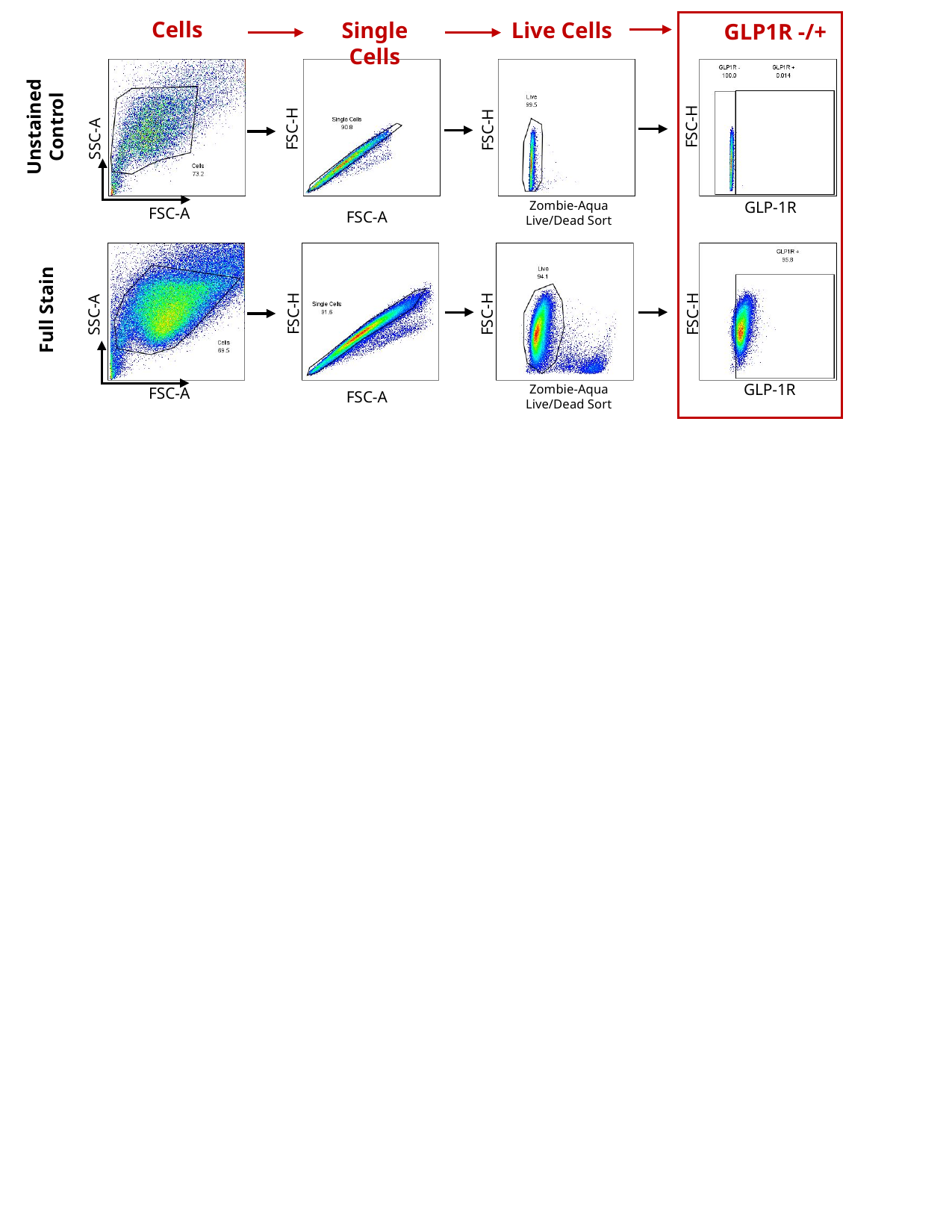

Cells
Live Cells
Single Cells
GLP1R -/+
Unstained Control
FSC-H
FSC-H
FSC-H
SSC-A
Zombie-Aqua
Live/Dead Sort
GLP-1R
FSC-A
FSC-A
Full Stain
FSC-H
FSC-H
SSC-A
FSC-H
GLP-1R
Zombie-Aqua
Live/Dead Sort
FSC-A
FSC-A

## Slide 2
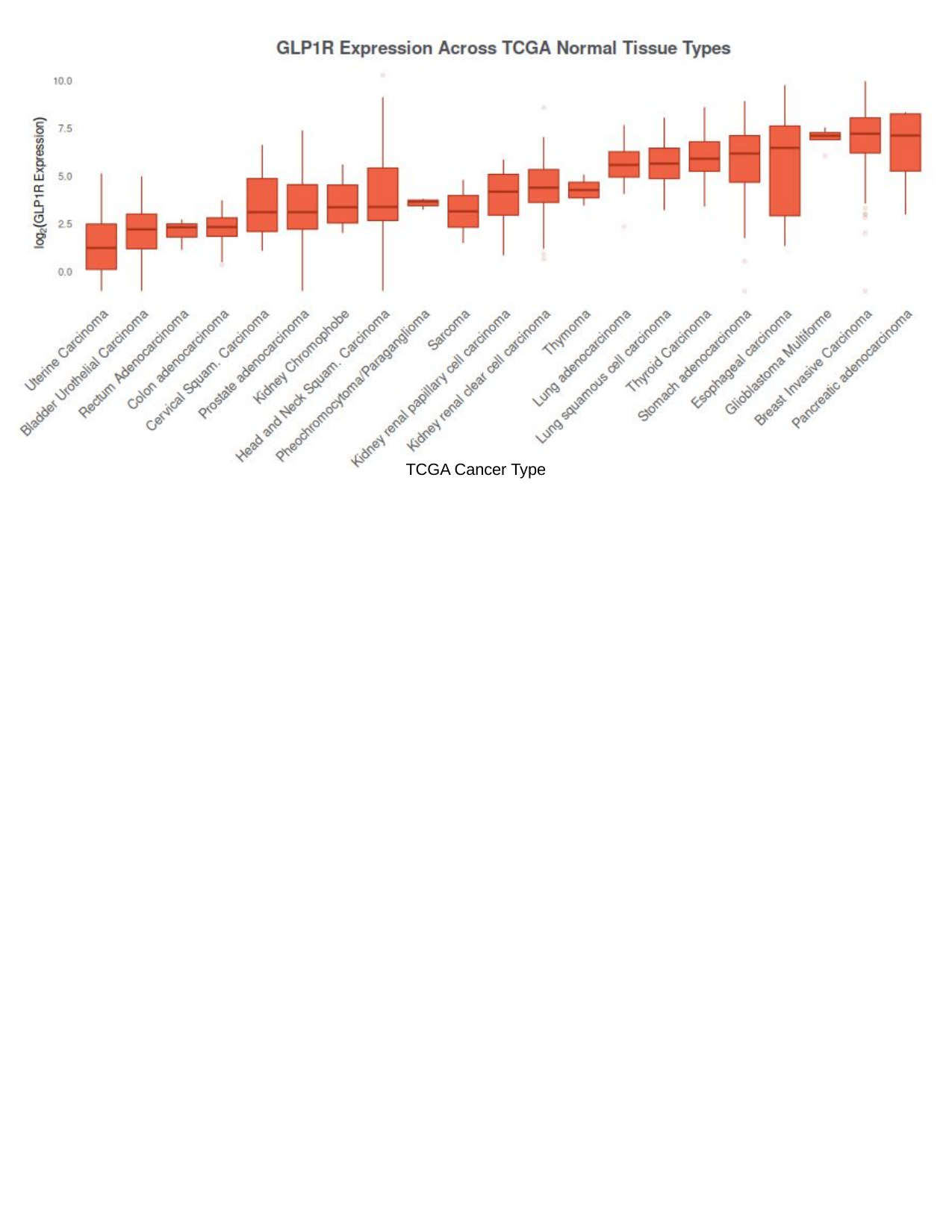

TCGA Cancer Type

## Slide 3
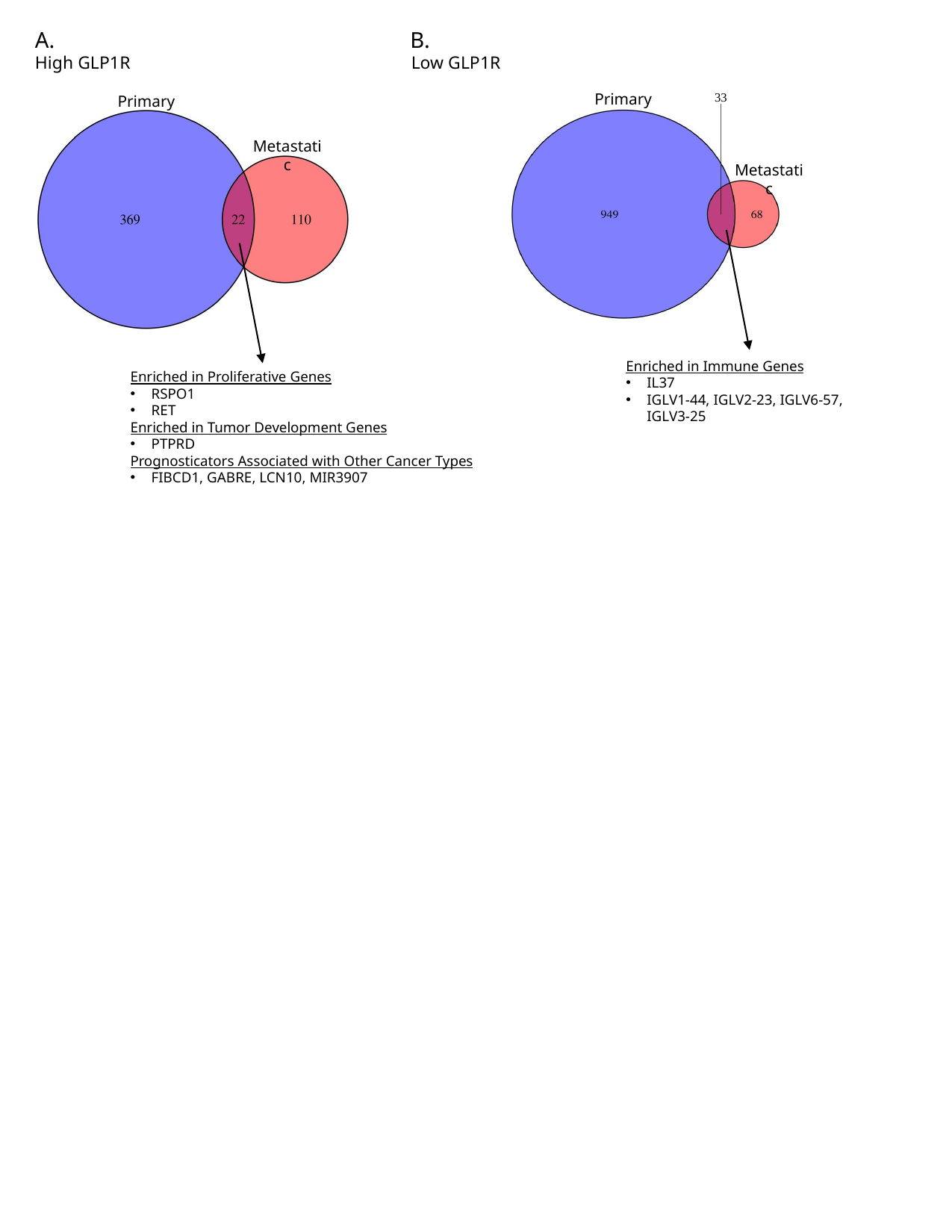

A. 			 B.
High GLP1R                  	 Low GLP1R
Primary
33
Primary
Metastatic
Metastatic
Enriched in Immune Genes
IL37
IGLV1-44, IGLV2-23, IGLV6-57, IGLV3-25
Enriched in Proliferative Genes
RSPO1
RET
Enriched in Tumor Development Genes
PTPRD
Prognosticators Associated with Other Cancer Types
FIBCD1, GABRE, LCN10, MIR3907
