# Supplementary material for: Glucagon-like peptide-1 receptor (GLP-1R) overexpression defines a distinct immunogenetic subset in primary and metastatic thyroid cancer: implications for GLP-1R agonist therapy
Source: Front Oncol. 2026 May 28;16:1834606. doi: 10.3389/fonc.2026.1834606 (PMC13253391; doi:10.3389/fonc.2026.1834606)
Supplement: Supplementary file 2 [file Supplementaryfile1.ppt]

## Slide 1
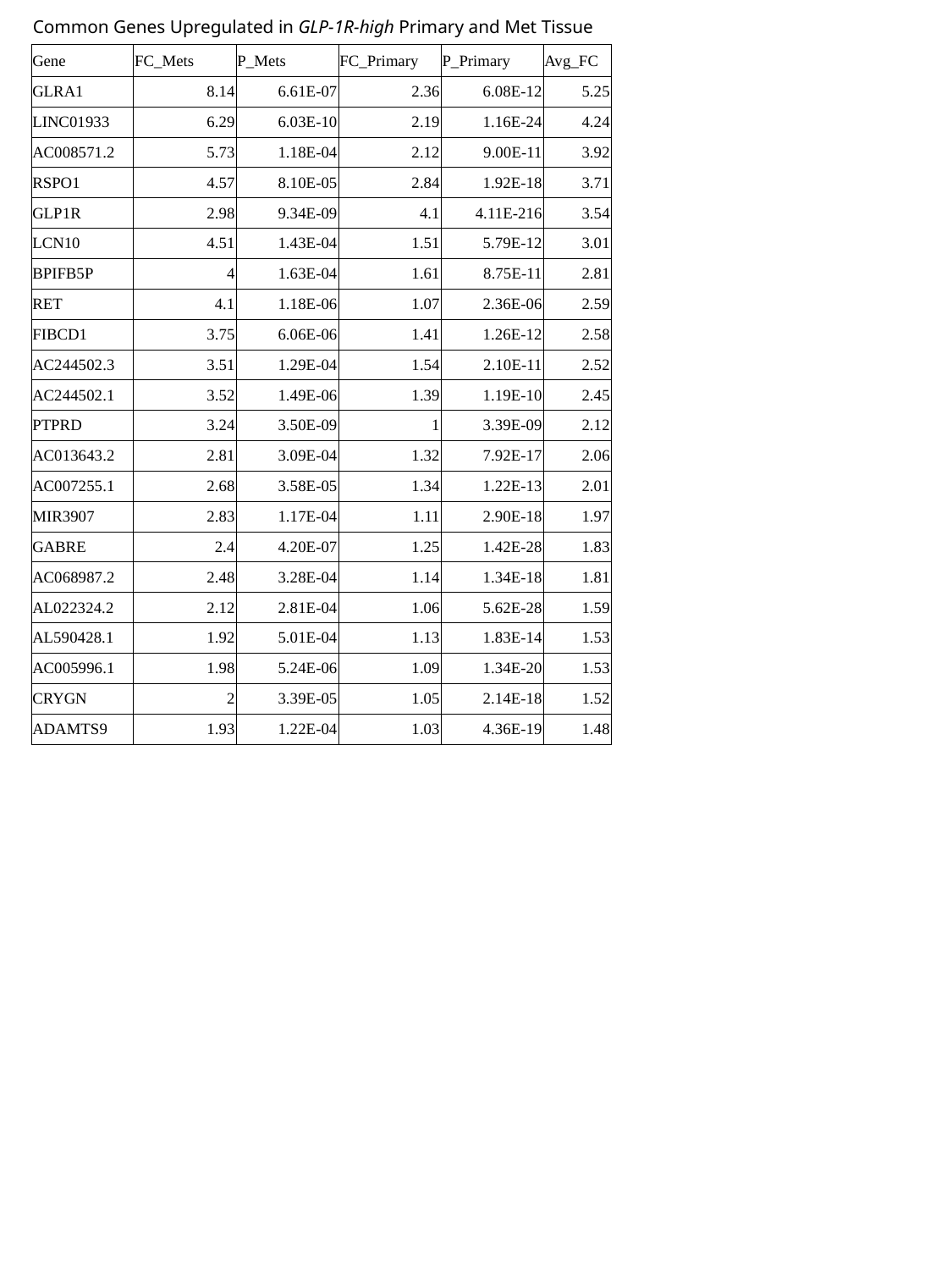

Common Genes Upregulated in GLP-1R-high Primary and Met Tissue
| Gene | FC\_Mets | P\_Mets | FC\_Primary | P\_Primary | Avg\_FC |
| --- | --- | --- | --- | --- | --- |
| GLRA1 | 8.14 | 6.61E-07 | 2.36 | 6.08E-12 | 5.25 |
| LINC01933 | 6.29 | 6.03E-10 | 2.19 | 1.16E-24 | 4.24 |
| AC008571.2 | 5.73 | 1.18E-04 | 2.12 | 9.00E-11 | 3.92 |
| RSPO1 | 4.57 | 8.10E-05 | 2.84 | 1.92E-18 | 3.71 |
| GLP1R | 2.98 | 9.34E-09 | 4.1 | 4.11E-216 | 3.54 |
| LCN10 | 4.51 | 1.43E-04 | 1.51 | 5.79E-12 | 3.01 |
| BPIFB5P | 4 | 1.63E-04 | 1.61 | 8.75E-11 | 2.81 |
| RET | 4.1 | 1.18E-06 | 1.07 | 2.36E-06 | 2.59 |
| FIBCD1 | 3.75 | 6.06E-06 | 1.41 | 1.26E-12 | 2.58 |
| AC244502.3 | 3.51 | 1.29E-04 | 1.54 | 2.10E-11 | 2.52 |
| AC244502.1 | 3.52 | 1.49E-06 | 1.39 | 1.19E-10 | 2.45 |
| PTPRD | 3.24 | 3.50E-09 | 1 | 3.39E-09 | 2.12 |
| AC013643.2 | 2.81 | 3.09E-04 | 1.32 | 7.92E-17 | 2.06 |
| AC007255.1 | 2.68 | 3.58E-05 | 1.34 | 1.22E-13 | 2.01 |
| MIR3907 | 2.83 | 1.17E-04 | 1.11 | 2.90E-18 | 1.97 |
| GABRE | 2.4 | 4.20E-07 | 1.25 | 1.42E-28 | 1.83 |
| AC068987.2 | 2.48 | 3.28E-04 | 1.14 | 1.34E-18 | 1.81 |
| AL022324.2 | 2.12 | 2.81E-04 | 1.06 | 5.62E-28 | 1.59 |
| AL590428.1 | 1.92 | 5.01E-04 | 1.13 | 1.83E-14 | 1.53 |
| AC005996.1 | 1.98 | 5.24E-06 | 1.09 | 1.34E-20 | 1.53 |
| CRYGN | 2 | 3.39E-05 | 1.05 | 2.14E-18 | 1.52 |
| ADAMTS9 | 1.93 | 1.22E-04 | 1.03 | 4.36E-19 | 1.48 |

## Slide 2
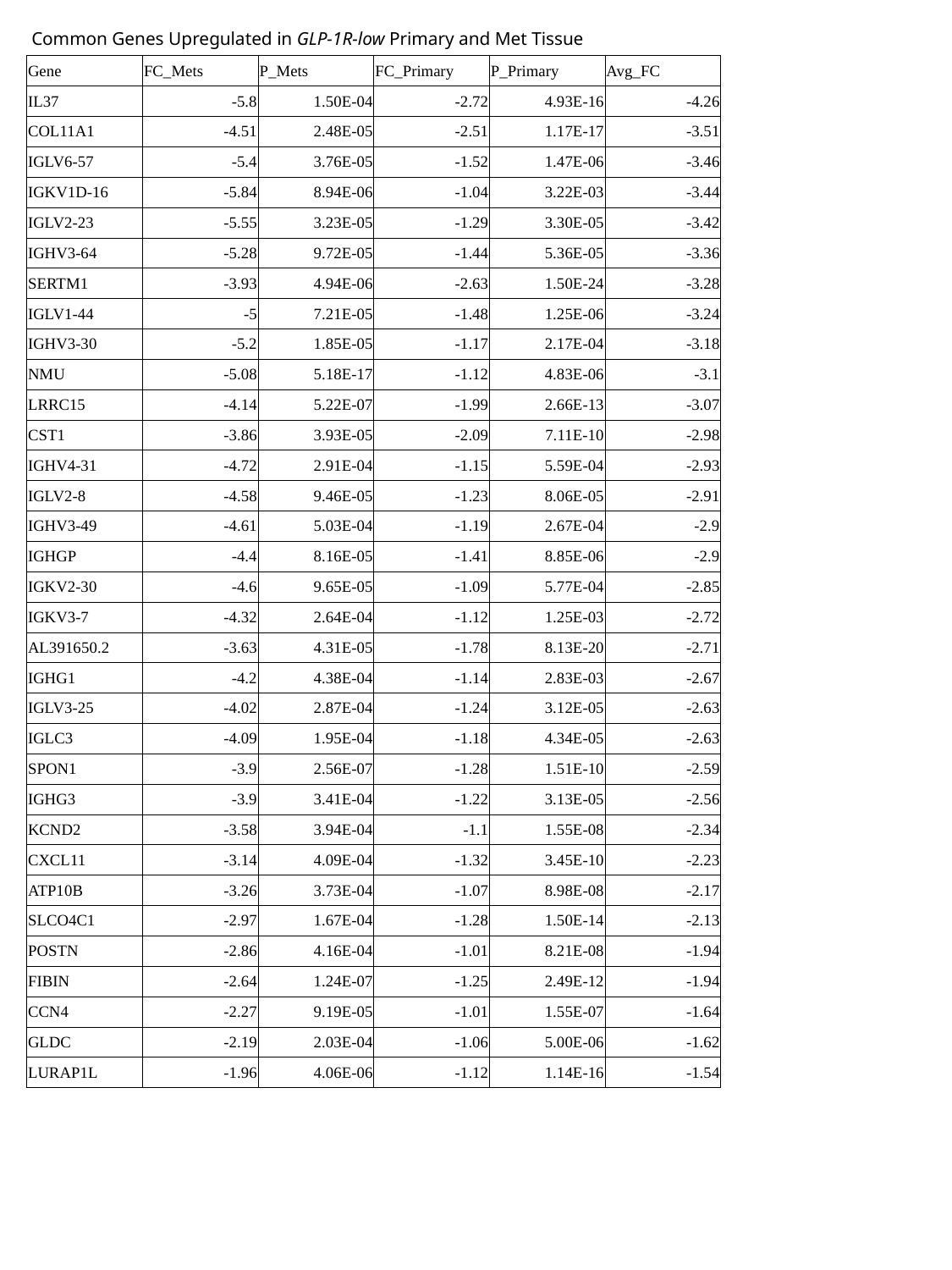

Common Genes Upregulated in GLP-1R-low Primary and Met Tissue
| Gene | FC\_Mets | P\_Mets | FC\_Primary | P\_Primary | Avg\_FC |
| --- | --- | --- | --- | --- | --- |
| IL37 | -5.8 | 1.50E-04 | -2.72 | 4.93E-16 | -4.26 |
| COL11A1 | -4.51 | 2.48E-05 | -2.51 | 1.17E-17 | -3.51 |
| IGLV6-57 | -5.4 | 3.76E-05 | -1.52 | 1.47E-06 | -3.46 |
| IGKV1D-16 | -5.84 | 8.94E-06 | -1.04 | 3.22E-03 | -3.44 |
| IGLV2-23 | -5.55 | 3.23E-05 | -1.29 | 3.30E-05 | -3.42 |
| IGHV3-64 | -5.28 | 9.72E-05 | -1.44 | 5.36E-05 | -3.36 |
| SERTM1 | -3.93 | 4.94E-06 | -2.63 | 1.50E-24 | -3.28 |
| IGLV1-44 | -5 | 7.21E-05 | -1.48 | 1.25E-06 | -3.24 |
| IGHV3-30 | -5.2 | 1.85E-05 | -1.17 | 2.17E-04 | -3.18 |
| NMU | -5.08 | 5.18E-17 | -1.12 | 4.83E-06 | -3.1 |
| LRRC15 | -4.14 | 5.22E-07 | -1.99 | 2.66E-13 | -3.07 |
| CST1 | -3.86 | 3.93E-05 | -2.09 | 7.11E-10 | -2.98 |
| IGHV4-31 | -4.72 | 2.91E-04 | -1.15 | 5.59E-04 | -2.93 |
| IGLV2-8 | -4.58 | 9.46E-05 | -1.23 | 8.06E-05 | -2.91 |
| IGHV3-49 | -4.61 | 5.03E-04 | -1.19 | 2.67E-04 | -2.9 |
| IGHGP | -4.4 | 8.16E-05 | -1.41 | 8.85E-06 | -2.9 |
| IGKV2-30 | -4.6 | 9.65E-05 | -1.09 | 5.77E-04 | -2.85 |
| IGKV3-7 | -4.32 | 2.64E-04 | -1.12 | 1.25E-03 | -2.72 |
| AL391650.2 | -3.63 | 4.31E-05 | -1.78 | 8.13E-20 | -2.71 |
| IGHG1 | -4.2 | 4.38E-04 | -1.14 | 2.83E-03 | -2.67 |
| IGLV3-25 | -4.02 | 2.87E-04 | -1.24 | 3.12E-05 | -2.63 |
| IGLC3 | -4.09 | 1.95E-04 | -1.18 | 4.34E-05 | -2.63 |
| SPON1 | -3.9 | 2.56E-07 | -1.28 | 1.51E-10 | -2.59 |
| IGHG3 | -3.9 | 3.41E-04 | -1.22 | 3.13E-05 | -2.56 |
| KCND2 | -3.58 | 3.94E-04 | -1.1 | 1.55E-08 | -2.34 |
| CXCL11 | -3.14 | 4.09E-04 | -1.32 | 3.45E-10 | -2.23 |
| ATP10B | -3.26 | 3.73E-04 | -1.07 | 8.98E-08 | -2.17 |
| SLCO4C1 | -2.97 | 1.67E-04 | -1.28 | 1.50E-14 | -2.13 |
| POSTN | -2.86 | 4.16E-04 | -1.01 | 8.21E-08 | -1.94 |
| FIBIN | -2.64 | 1.24E-07 | -1.25 | 2.49E-12 | -1.94 |
| CCN4 | -2.27 | 9.19E-05 | -1.01 | 1.55E-07 | -1.64 |
| GLDC | -2.19 | 2.03E-04 | -1.06 | 5.00E-06 | -1.62 |
| LURAP1L | -1.96 | 4.06E-06 | -1.12 | 1.14E-16 | -1.54 |
